# Supplementary material for: The Link of mRNA and rRNA Transcription by PUF60/FIR through TFIIH/P62 as a Novel Therapeutic Target for Cancer
Source: Int J Mol Sci. 2023 Dec 11;24(24):17341. doi: 10.3390/ijms242417341 (PMC10743661; doi:10.3390/ijms242417341)
Supplement: Supplementary file 1 [file ijms-24-17341-s001.zip › Kitamura_Supplemental Fig. and Table legends.pdf]

Supplemental Fig Legends and Supplemental Table titles

Supplemental Fig. S1. Original gels/blots of western blot used in this study. a Original gels/blots of western blot used in Figure 3a. **b** The cropped gels/blots were indicated in red line used in Figure 5a. **c** High-contrast (overexposure) gel used is Figure 5b. **d** Original gels/blots of western blot used in Figure 5c.

Supplemental Fig. S2. The mRNA expression of rRNAs affected by FIR and FIRΔexon2. The expression of rRNAs is analyzed by qRT-PCR in HCT116 cells. **a** The mRNA expression of *RPL19*, *RPL23A*, *RPL30*, *RPL36*, *RPL37*, *RPL37A*, *RPL38*, *RPS6*, *RPS10*, *RPS14*, *RPS15A*, *RPS16*, *RPS19*, *RPS21*, and *RPS29* was examined by qRT-PCT in HCT116 cells under the overexpression of FIR and FIRΔexon2. **b** The mRNA expression of *RPL19*, *RPL23A*, *RPL30*, *RPL36*, *RPL37*, *RPL37A*, *RPL38*, *RPS6*, *RPS10*, *RPS14*, *RPS15A*, *RPS16*, *RPS19*, *RPS21*, and *RPS29* was examined by qRT-PCT in HCT116 cells under the knockdown of FIR and FIRΔexon2 by siRNA.

Supplemental Fig. S3. The mRNA expression of rRNA by overexpression or knockdown of FIR or FIRΔexon2 in human cell lines. a Over expression FIR or FIRΔexon2 affected the mRNA expression of *RPS21* in HeLa cells. **b** Over expression FIR-FLAG or FIRΔexon2-FLAG affected the mRNA expression of *RPS29* in HeLa cells. **c** Knockdown of FIRΔexon2 by siRNA

affected the mRNA expression of *RPS15A* in HeLa cells. **d** The mRNA expression of *RPS21, RPS29,* and *RPS15A* was significantly decreased by both the knockdown of FIR and FIRΔexon2 by siRNA in HepG2 cells. **e** The expression of FIR was examined by the knockdown of FIR and FIRΔexon2 by siRNA in T98G cells (Western blot, duplicate). b-actin was used for an internal control. **f** The expression of FIR and FIRΔexon2 mRNA was examined by the knockdown of FIR and FIRΔexon2 by siRNA in T98G cells (qRT-PCR, duplicate). b-actin was used for an internal control. **g** The mRNA expression of *RPS21*, *RPS29* and *RPS15A* by the knockdown of FIR or FIRΔexon2 by siRNA in T98G cells.

Supplemental Fig. S4. rDNAs or genes for rRNA affected by FIR and FIRDexon2 were located on specific chromosomes a The number of chromosomes is indicated that upregulated differentially expressed genes (DGEs) by overexpression of FIR-FLAG and knockdown of FIR by siRNA. Among 66 affected genes for rRNA, 48 genes were on chromosomes 17, 19, 1,12, 5,16, 2, 6, 9, and 11; 72.7% (48/66) among top 10 chromosomes (square). **b** The number of chromosomes is indicated that DGEs by overexpression of FIRΔexon2-FLAG and knockdown of FIRΔexon2 by siRNA. Among 100 affected genes for rRNA, 70 genes were on chromosomes 17, 1, 12, 6, 19, 5, 2, 9, 11, and 3, that is 70.0% (70/100) among top 10 chromosomes (square).

Supplemental Fig. S5. Structure of PUF60/FIR gene and pathogenic variants reported in human diseases. a The amino sequence of FIR (542aa) FIRΔexon2 (513aa) is indicated. Exons are indicated by different colors. Exon 2 is indicated in red. RRM1; magenta, RRM2; yellow, and RRM3/UHM; blue. For the structures of the regions colored in gray, it is not determined where the P62-binding sequence exists. Amino acids in red square show RPB6 homology sequence. **b** Pathogenic variants of PUF60/FIR were found in congenital diseases, Verheij syndrome and CHARGE syndrome. These pathogenic variants were reported in the literature (listed in supplemental table 6) that generated truncated FIR protein lacking the P62-binding site and RRM3/UHM. **c** Dimer formation of two FIR proteins with RRM1 and RRM2 observed in the crystal structure of 2QFJ (see test). **c** RRM1 and RRM2 domain of FIR interact with ssDNA. Two FIR protein binds to ssDNA of FUSE located at c-myc promoter.

Table S1. Screening of FIR interacting proteins by yeast-two hybrid analysis.

Table S2. Genes for rRNA affected by overexpression or knockdown of FIR or FIRΔexon2 (HeLa cells).

Table S3. List of DEGs associated with mTOR/p70S6K/EIF signaling pathway by alteration of FIR or FIRΔexon2.

Table S4. Structures of small molecular weight chemicals that were interacted with His-FIR or His-FIRDexon2 screened by NPDepo.

Table S5. Pathologic variants of *FIR/PUF60* in human hereditary disease reported in the literature.

Table S6. List of antibodies, siRNA and primers used in this study.
