# Supplementary figures and images for: The Link of mRNA and rRNA Transcription by PUF60/FIR through TFIIH/P62 as a Novel Therapeutic Target for Cancer
Source: Int J Mol Sci. 2023 Dec 11;24(24):17341. doi: 10.3390/ijms242417341 (PMC10743661; doi:10.3390/ijms242417341)

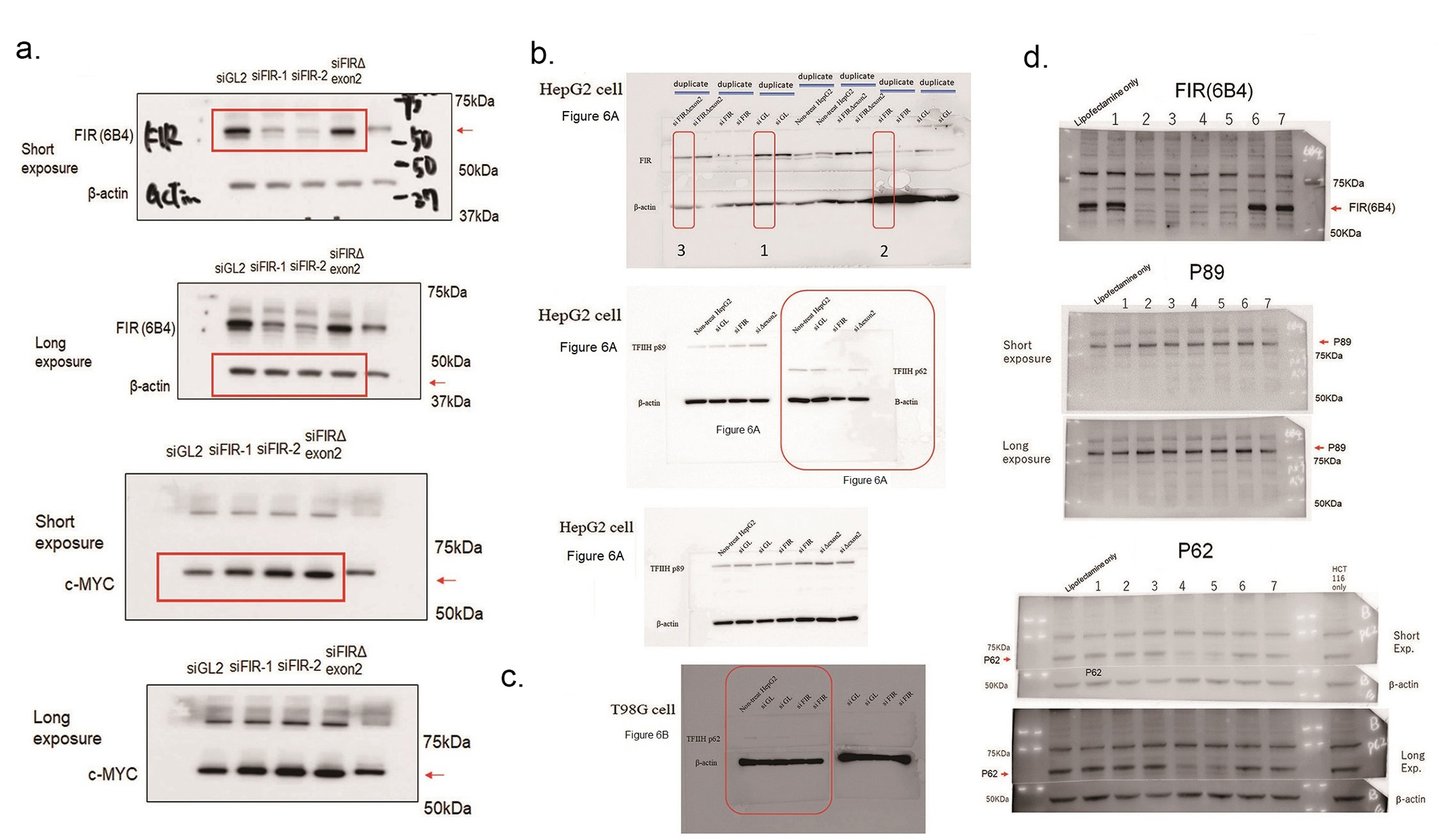

Supplement: Supplementary file 1 [file ijms-24-17341-s001.zip › Kitamura K et al Supplementary Figure S1.png]

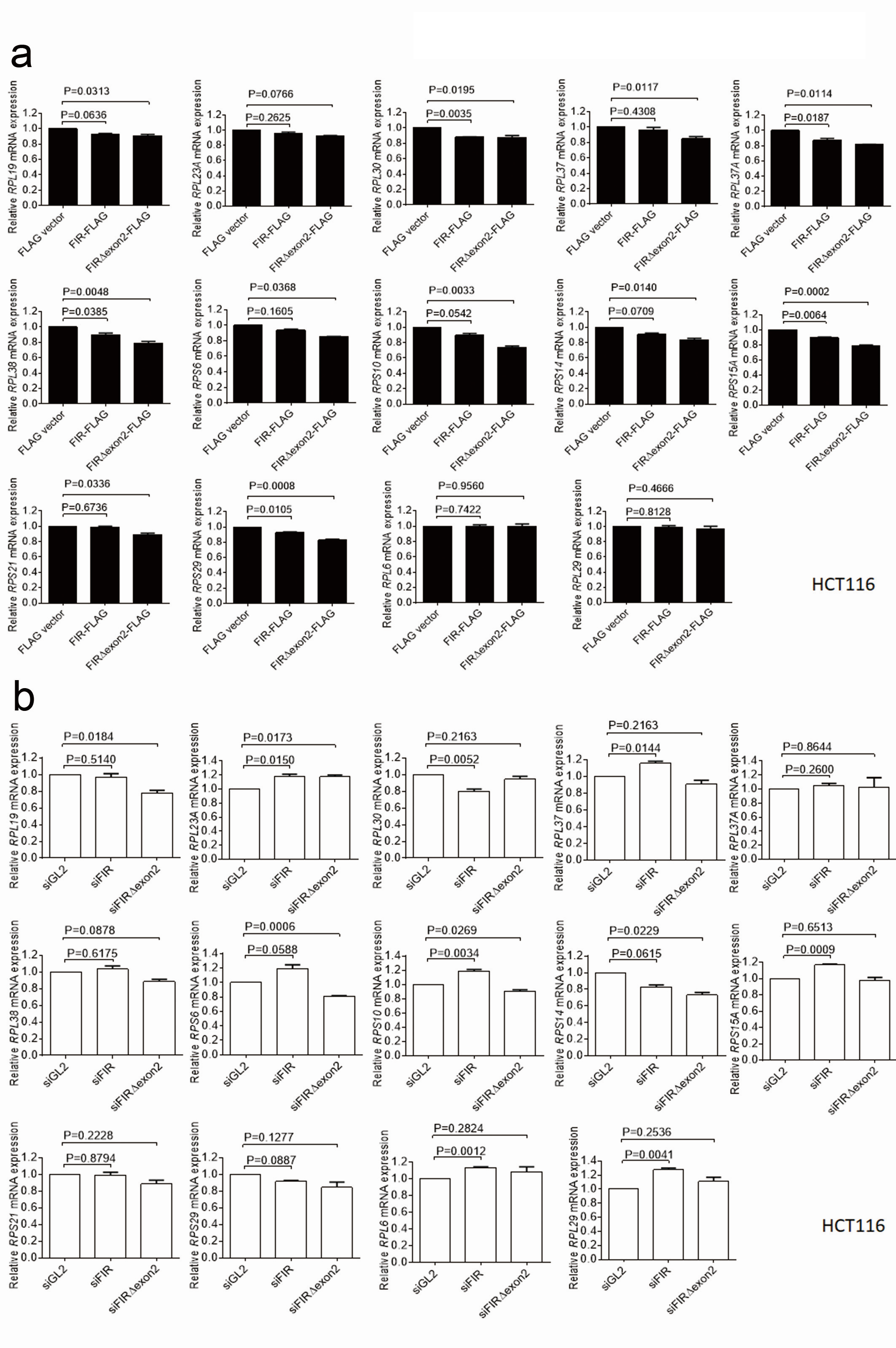

Supplement: Supplementary file 1 [file ijms-24-17341-s001.zip › Kitamura K et al Supplementary Figure S2.png]

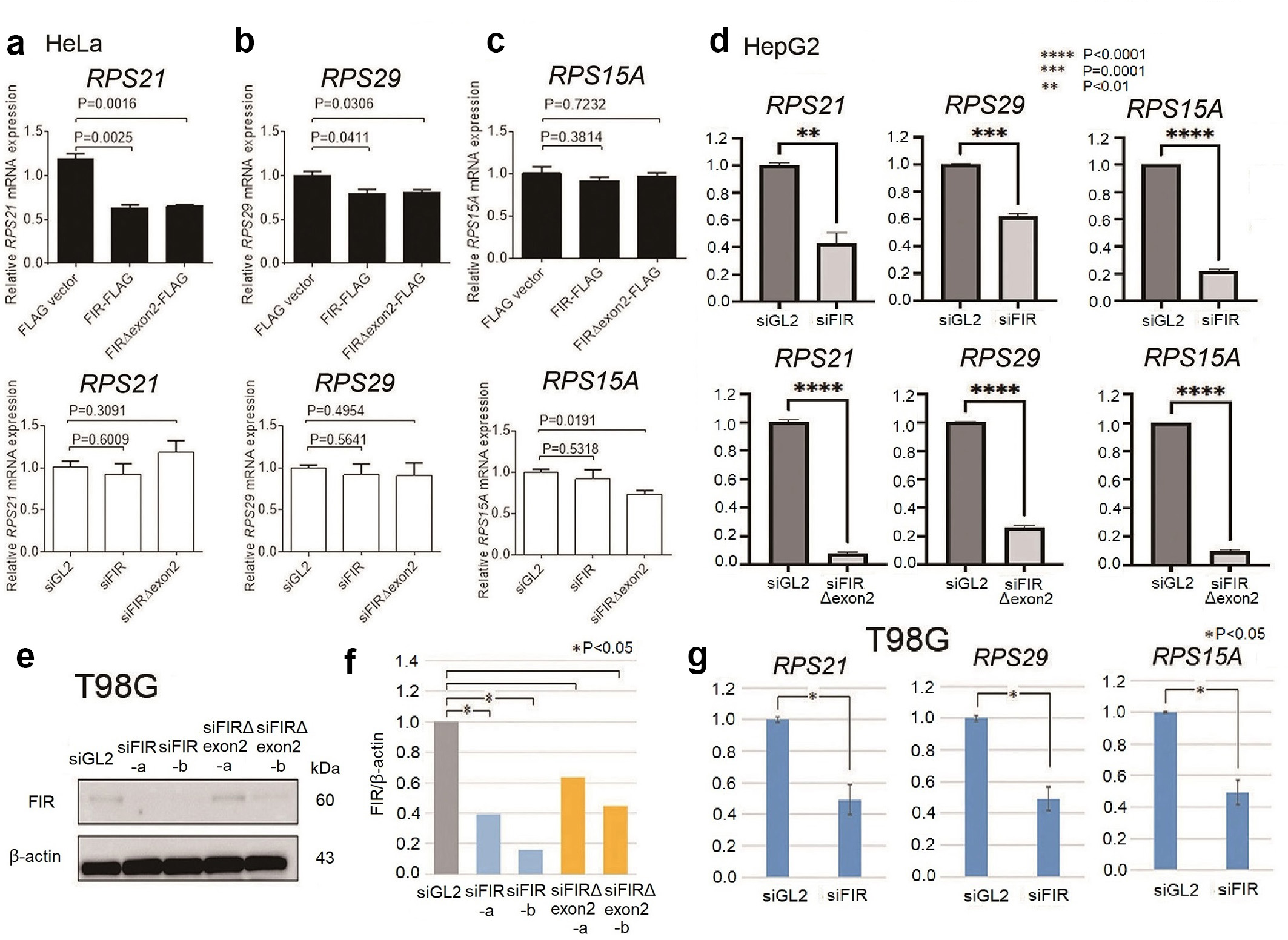

Supplement: Supplementary file 1 [file ijms-24-17341-s001.zip › Kitamura K et al Supplementary Figure S3.png]

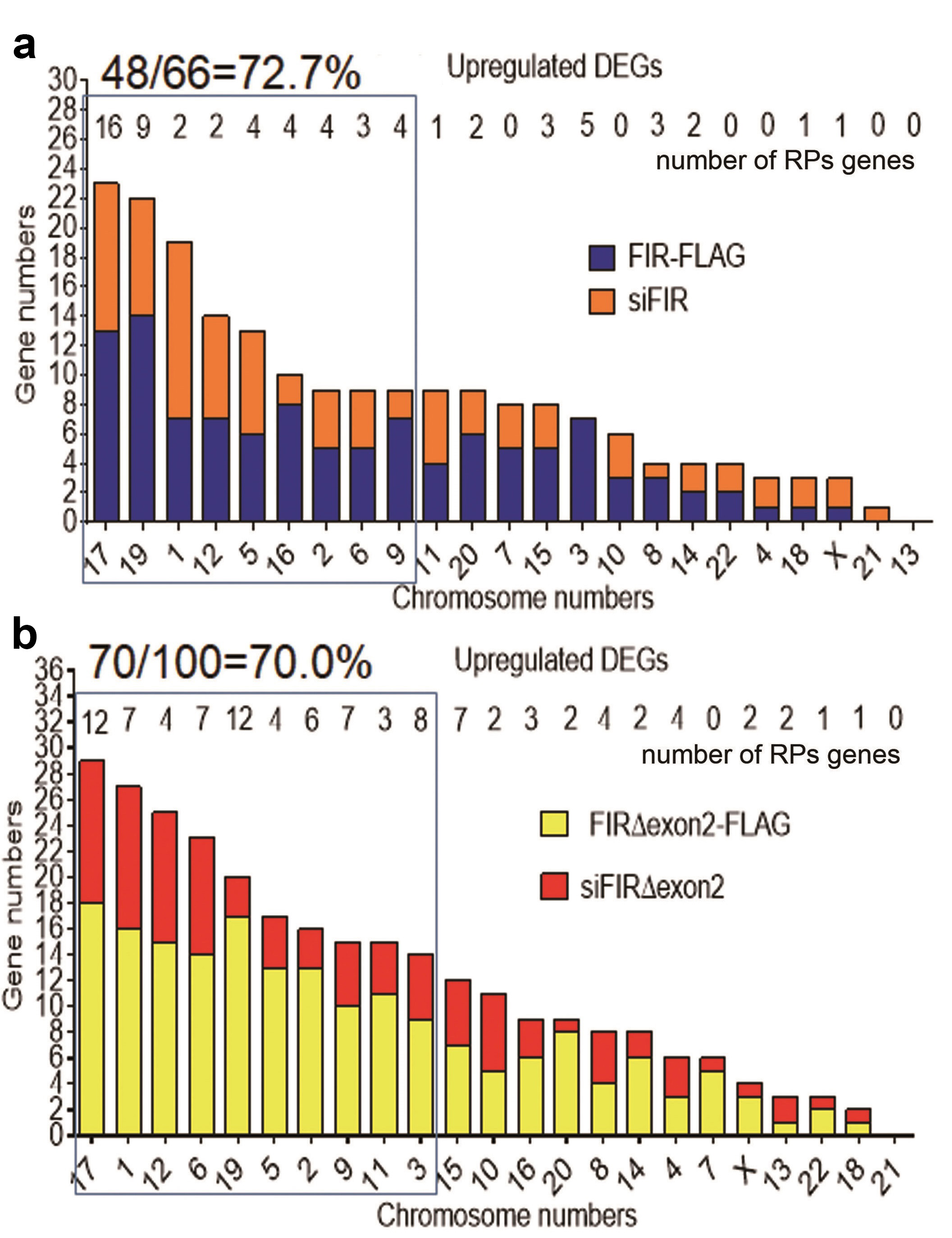

Supplement: Supplementary file 1 [file ijms-24-17341-s001.zip › Kitamura K et al Supplementary Figure S4.png]

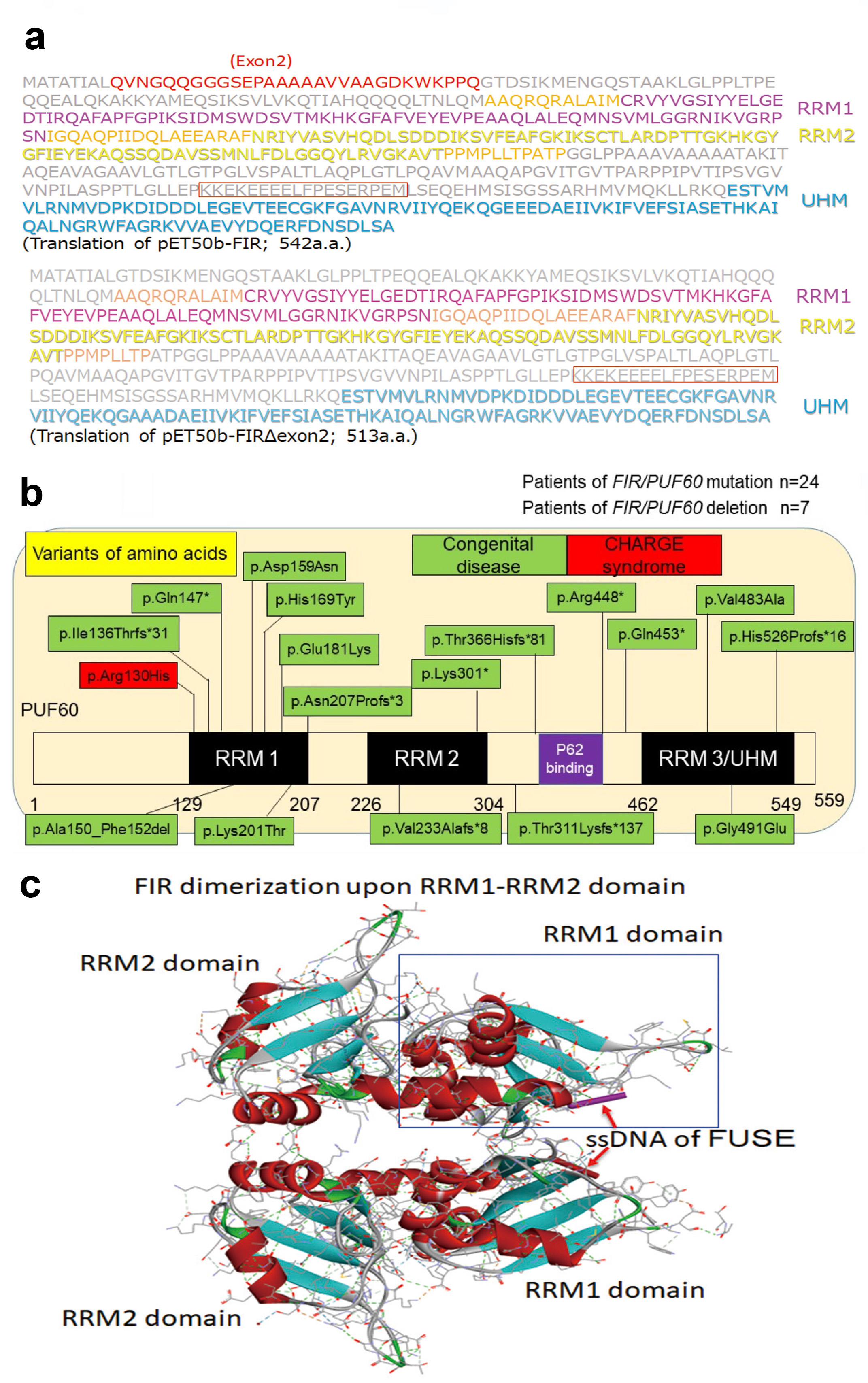

Supplement: Supplementary file 1 [file ijms-24-17341-s001.zip › Kitamura K et al Supplementary Figure S5.png]
